# Inadvertent Transfer of Murine VL30 Retrotransposons to CAR-T Cells

**DOI:** 10.1155/2022/6435077

**Published:** 2022-05-31

**Authors:** Sung Hyun Lee, Yajing Hao, Tong Gui, Gianpietro Dotti, Barbara Savoldo, Fei Zou, Tal Kafri

**Affiliations:** 1 Gene Therapy Center, University of North Carolina at Chapel Hill, Chapel Hill, North Carolina, USA; 2 Department of Biostatistics, University of North Carolina, Chapel Hill, North Carolina, USA; 3 Department of Microbiology and Immunology, University of North Carolina at Chapel Hill, Chapel Hill, North Carolina, USA; 4 Lineberger Comprehensive Cancer Center, University of North Carolina at Chapel Hill, Chapel Hill, North Carolina, USA; 5 Department of Pediatrics, University of North Carolina at Chapel Hill, Chapel Hill, North Carolina, USA; 6 Department of Genetics, University of North Carolina, Chapel Hill, North Carolina, USA

## Abstract

For more than a decade, genetically engineered autologous T-cells have been successfully employed as immunotherapy drugs for patients with incurable blood cancers. The active components in some of these game-changing medicines are autologous T-cells that express viral vector-delivered chimeric antigen receptors (CARs), which specifically target proteins that are preferentially expressed on cancer cells. Some of these therapeutic CAR expressing T-cells (CAR-Ts) are engineered via transduction with *γ*-retroviral vectors (*γ*-RVVs) produced in a stable producer cell line that was derived from murine PG13 packaging cells (ATCC CRL-10686). Earlier studies reported on the copackaging of murine virus-like 30S RNA (VL30) genomes with *γ*-retroviral vectors generated in murine stable packaging cells. In an earlier study, VL30 mRNA was found to enhance the metastatic potential of human melanoma cells. These findings raise biosafety concerns regarding the possibility that therapeutic CAR-Ts have been inadvertently contaminated with potentially oncogenic VL30 retrotransposons. In this study, we demonstrated the presence of infectious VL30 particles in PG13 cell-conditioned media and observed the ability of these particles to deliver transcriptionally active VL30 genomes to human cells. Notably, VL30 genomes packaged by HIV-1-based vector particles transduced naïve human cells in culture. Furthermore, we detected the transfer and expression of VL30 genomes in clinical-grade CAR-T cells generated by transduction with PG13 cell-derived *γ*-retroviral vectors. Our findings raise biosafety concerns regarding the use of murine packaging cell lines in ongoing clinical applications.

## 1. Introduction

Following successful clinical trials, the FDA and, later, the European, Canadian, and Swiss regulatory administrations approved a gene therapy-based immunotherapy drug for adult patients with diffuse large B-cell lymphoma (DLBCL) who relapsed or did not respond to two conventional anticancer treatments [1–3]. The abovementioned medicine comprises autologous T-cells that were transduced *in vitro* with *γ*-retroviral vectors (*γ*-RVVs) expressing CARs directed to the B cell-specific protein CD19. Production of the abovementioned therapeutic viral vectors is premised on a stable producer cell line derived from murine PG13 packaging cells (ATCC CRL-10686) [4–6]. Reproducibility in the quality and quantity of vector preparations and the ability to scale up *γ*-RVV production were the impetus for the development of various stable packaging cell lines, most of which were derived from NIH 3T3 fibroblast cells [7]. Active murine endogenous retroviruses (ERVs) [8–10] raise biosafety concerns associated with the possibility that murine LTR retrotransposons may be copackaged along with clinical-grade *γ*-RVVs [11]. Indeed, earlier studies reported on efficient packaging of the murine VL30 retrotransposon into *γ*-RVV particles generated in various murine packaging cells [12–17]. Furthermore, a study by Song et al. demonstrated the ability of the VL30 genome to enhance the metastatic potential of human melanoma cells in immunodeficient mice [18]. In an earlier study, Purcell et al. detected VL30 genomes in lymphoma cells in nonhuman primates transplanted with *γ*-RVV-transduced hematopoietic stem cells [16]. VL30 genomes do not include protein-encoding open-reading frames [18, 19]. However, the VL30 mRNA functions as a long noncoding RNA (lncRNA), which efficiently binds the murine and the human tumor suppressor protein PTB-associated splicing factor (PFS) [18, 20–22]. This protein is involved in multiple cellular pathways including DNA repair, RNA processing, and regulation of the innate immune response [23–27]. We consider the possibility that inadvertent infection of human cells with VL30 retrotransposons can potentially mediate insertional mutagenesis, induce oncogenic pathways, and contribute to the emergence of novel pathogens [28]. Importantly, to date, the PG13 packaging cell line has not been characterized for retroelement secretion and the FDA-required quality control analysis of CAR-Ts does not include testing for the presence of endogenous retroelement genomes at various stages of CAR-T production. In this study, for the first time, we characterized the secretion of VL30 genome-containing *γ*-RVV particles by PG13 packaging cells. Our findings indicate that murine VL30 genomes were efficiently delivered, reverse transcribed, and expressed in human 293T cells following exposure to conditioned media from PG13 cells. VL30 genomes that integrated into the chromatin of human cells were mobilized and transferred by HIV-based vectors to naïve human cells in culture. Furthermore, we detected transcriptionally active VL30 genomes in CAR-Ts generated by a clinical-grade protocol. Our findings raise biosafety concerns regarding the ongoing and past usage of murine packaging cell lines in clinical gene therapy applications.

## 2. Materials and Methods

### 2.1. Cells.

The PG13 *γ*-retroviral vector packaging cell line was purchased from the American Type Culture Collection (CRL-10686, ATCC, Manassas, VA). PG13 and 293T cells were cultured in Dulbecco’s modified Eagle’s medium (DMEM) high glucose (10-013-CV, Corning Mediatech Inc., Manassas, VA) with 1x antibiotic antimycotic (15240-062, Gibco, Thermo Fisher Scientific, Grand Island, NY) and 10% fetal bovine serum (10437-028, Gibco, Thermo Fisher Scientific, Grand Island, NY). Peripheral blood mononuclear cells (PBMCs) were isolated from buffy coats (Gulf Coast Regional Blood Center) using Lymphoprep (AN1001967, Accurate Chemical and Scientific Corporation, Carle Place, NY). Next, PBMCs were activated using 1 *μ*g/mL immobilized CD3 and CD28 antibodies (Miltenyi Biotec, Cambridge, MA). After 3 days, cells were transduced in 24-well plates precoated with recombinant fibronectin (FN CH-296, RetroNectin, TaKaRa, Clontech, Mountain View, CA) with retroviral supernatants and expanded in complete medium (45% RPMI-1640 and 45% Click’s medium, 10% FBS, and 2 mM GlutaMAX) supplemented with IL-7 (10 ng/mL, Miltenyi) and IL-15 (5 ng/mL, Miltenyi). After transduction, T-cells were expanded ex vivo in complete medium (45% RPMI-1640 and 45% Click’s medium, 10% FBS, and 2 mM GlutaMAX) in the presence of IL-7 (10 ng/mL) and IL-15 (5 ng/mL), which were added twice a week for up to 15 days.

### 2.2. Isolation of Single-Cell 293T Clones Containing VL30 Genome.

293T cells were transduced with the lentiviral vector pTK1261, from which the firefly luciferase cDNA and the fusion GFP/blasticidin marker gene were expressed under the control of a CMV promoter and the encephalomyelitis virus internal ribosome entry site (IRES), respectively. Transduced cells (293T-1261) were selected for blasticidin resistance in the presence of blasticidin (5 *μ*g/mL). Blasticidin-resistant 293T cells were cocultured with PG13 cells. At confluency, the coculture (293T-1261/PG13) cell population was passaged and PG13 cells were eliminated in the presence of 5 *μ*g/mL blasticidin. The abovementioned process of coculturing was repeated seven times, after which single-cell clones of blasticidin-resistant 293T cells were isolated. The absence of PG13 cells was confirmed by the lack of PCR amplification of the mouse GAPDH gene. GCN in the abovementioned isolated single-cell clones was determined by qPCR as described as follows.

### 2.3. Production CAR Carrying *γ*-RVV from a Newly Established Stable Producer Cell Line.

A stock of Eco-pseudotyped SFG.iC9.GD2.CAR.IL15 *γ*-RVV was produced by transient transfection of the ecotropic *Φ*NX-Eco packaging cell line (American Type Culture Collection product CRL-3214; ATCC, Manassas, VA) with the vector expression cassette. A heterogenous stable producer cell line was generated by repeated transduction of the murine gibbon ape leukemia virus (GalV) envelope-expressing PG13 packaging cell line (# CRL-10686^™^, ATCC, Manassas, VA) with the abovementioned Eco-pseudotyped SFG.iC9.GD2 vector. To enrich for stable producer cells with high VCN, the abovementioned heterogenous population of vector-producing cells was immunostained with an anti-idiotype antibody specific for the GD2 CAR [29] and high-transgene expressing cells were sorted using the BD Jazz cell sorter. Following limiting dilution, single-cell clones of vector-producing cells were isolated and functionally screened for the highest biological titer by qPCR. The highest vector-producing clone was expanded for the generation of the Master Cell Bank (MCB) for the production of GalV envelope pseudotyped retroviral particles carrying the iC9. GD2.CAR.IL15 expression cassette. For each lot of *γ*-RVV supernatant, cells from the abovementioned MCB were thawed and expanded in the iCellis Nano bench-top bioreactor (Pall Inc.). The iCellis Nano is a fixed-bed bioreactor using fiber material for the attachment of adherent vector producer cells. The system was employed to provide continuous monitoring of dissolved oxygen, pH, and temperature. The system provided continuous circulation of culture media along with a proper gas mixture of O_2_ and CO_2_. pH was additionally controlled by the addition of base as required. After seeding onto the fiber bed of the iCellis Nano, the cells are cultured for 2–3 days to reach near confluency. At this point, the media was replaced with fresh media, and following overnight culture, the supernatant was harvested into a transfer bag and fresh media was added to the system. This supernatant is termed “day 1” harvest. This process was repeated four additional times resulting in a total of five days of supernatant harvest. After harvest of the fifth supernatant, the producer cells were removed from the bioreactor using enzymatic digestion (TrypLE) and washed with PBS. The harvested cells were counted and samples were taken for QC studies. The MCB, end of production cells, and viral supernatant were tested following FDA recommendations for sterility and absence of replication competent retroviral particles.

### 2.4. Lentiviral and *γ*-Retroviral Vectors.

The construction of the non-SIN *γ*-RVV SFG.iC9.GD2.CAR.IL15 was described earlier [29], pTK1261 was constructed by cloning a BglII/BamHI DNA fragment containing the firefly luciferase cDNA into a BamHI site in the lentiviral vector pTK642 [30], and pTK2229 was constructed by cloning an AfeI/HpaI DNA fragment comprising a VL30 sequence from nucleotides 2140 to 2769 of GenBank: AF486451.1 in a PshAI site in a lentiviral vector comprising a CMV promoter-regulated expression cassette (encoding the mCherry-T2A-Puromycin selection marker) in an opposite orientation to the LTRs. The pTK1808 and pTK2151 vectors were purchased from Addgene (Addgene, Watertown, cat# 14088 and 10668, respectively). Both vectors are premised on the *γ*-retroviral vector pBabe-puro and carry either the SV40 large T antigen or the green fluorescence protein (GFP) cDNA under the control of the vector 5′ LTR, respectively. Note that downstream to the SV40 large T antigen cDNA, the pTK1808 vector also contains the puromycin-resistant cDNA under the control of the SV40 promoter.

### 2.5. Production of Viral Vectors by Transient Transfection.

All vector particles were VSV-G pseudotyped and produced in 293T cells using the transient three-plasmid calcium phosphate transfection method as described earlier [31, 32]. In brief, the second-generation lentiviral vector packaging cassette *Δ*NRF [33] or the MLV Gag/Pol expression cassette (a kind gift from Dr. Nikunj Somia at the University of Minnesota) was transiently transfected into 293T cells along with the VSV-G envelope expression cassette and the relevant vector construct. Vector particles in conditioned media were harvested ~60 hours after transfection and filtered through a 0.45 *μ*m syringe filter.

### 2.6. Analysis of the VL30 Genome and *γ*-Retroviral Vector Copy Number (GCN and VCN, Respectively).

Genomic DNA samples were extracted by the DNeasy Blood & Tissue Kit (69506, QIAGEN, Hilden, Germany). To eliminate potential contamination with carried-over transfected plasmid DNA, all genomic DNA samples (except DNA samples extracted from cultured human T-cells) were digested with the DpnI restriction enzyme.

GCN and VCN were determined by quantitative real-time polymerase chain reaction (qPCR) using the QuantStudio^™^ 3 System (Applied Biosystems, Thermo Fisher Scientific, Waltham, MA). To measure VL30 GCN, a reference cell population containing a single copy of a VL30 sequence was established (293T-2229 cells). Specifically, 293T cells were transduced with the lentiviral vector pTK2229 (at MOI < 0.001) and selected for puromycin resistance in the presence of 5 *μ*g/ml of puromycin (P8833, MilliporeSigma, St. Louis, MO). DNA samples extracted from the abovementioned heterogenous population of puromycin-resistant 293T cells served to establish a reference DNA standard curve to measure VL30 VCN by qPCR using a primer/probe set (Integrated DNA Technologies Inc., Coralville, Iowa) comprising a forward primer 5′-CCTTGACCAGAAGCCACTATG-3′, a reverse primer 5′-TCAGAGATTGGGACCCTGAA-3′, and a 6-FAM^™^-conjugated probe 5′-TGTAAGATGGCCTGCTTGTCTGCA-3′.

To measure the VCN of *γ*-RVVs (expressing either a CAR or the GFP cDNA), a reference cell population comprising a single copy of a *γ*-RVV genome was established (293T-1808 cells). Specifically, 293T cells were transduced with the *γ*-RVV l vector pTK1808 (at MOI < 0.001) and selected for puromycin resistance in the presence of 5 *μ*g/mL of puromycin (MilliporeSigma, St. Louis, MO). DNA samples extracted from the abovementioned heterogenous population of puromycin-resistant 293T cells served to establish a reference DNA standard curve to measure *γ-*RVV VCN by qPCR using a primer/probe set (Integrated DNA Technologies Inc., Coralville, Iowa) comprising a forward primer 5′-CGCTGACGGGTAGTCAATC-3′, a reverse primer 5′-GGGTACCCGTGTATCCAATAAA-3′, and a 6-FAM^™^ probe 5′-ACTTGTGGTCTCGCTGTTCCTTGG-3′. qPCR analysis of endogenous human RNaseP, which served as an internal reference control, was based on a commercial human RNaseP primer/probe set (443328, Applied Biosystems, Thermo Fisher Scientific, Waltham, MA).

PCR amplification of the mouse GAPDH gene was based on a primer/probe set from Hoffmann-La Roche Ltd., Basel, Switzerland (Universal ProbeLibrary Mouse GAPD Gene Assay, 05046211001). qPCR was performed with the ABsolute qPCR ROX Mix (AB-1138/B, Applied Biosystems, Thermo Fisher Scientific, Waltham, MA) under the following conditions: 95°C for 15 min and then 40 cycles of 95°C for 15 sec and 60°C for 1 min.

### 2.7. RNA Isolation and qRT-PCR.

RNA was isolated using an RNeasy^®^ Plus Mini Kit (QIAGEN, Hilden, Germany) and converted to cDNA using a QuantiTect^®^ Reverse Transcription Kit (QIAGEN, Hilden, Germany). qRT-PCR of VL30 and *γ*-RVV mRNA was based on the same primer/probe sets that were described. The qRT-PCR assay for human ACTB mRNA, which served as an internal reference control, was premised on a commercial set of primers/probes (Hs. PT.39a.22214847) conjugated with HEX^™^ at the 5′ end (Integrated DNA Technologies Inc., Coralville, Iowa). All PCR results were analyzed with Prism 9 software (GraphPad Software, San Diego, CA).

### 2.8. Statistical Analysis.

The VL30 genome copy number (GCN) and *γ*-RVV vector copy number (VCN) in human T-cells transduced with CAR-expressing *γ*-RVV were calculated as the average of 3 technical replicates. The association between VL30-GCN and *γ*-RVV in primary human CAR-T cells was estimated by Pearson correlation and tested with linear regression analysis.

Statistical analyses employed to characterize the significance of various treatments’ effects on VL30-GCN and *γ*-RVV-VCN are outlined in the figure legends.

### 2.9. Ethics Statement.

The study (# 21–0417) was reviewed by the UNC Office of Human Research Ethics: submission reference ID 322906. The review panel determined that the submission does not constitute human subject research and does not require IRB approval.

### 2.10. Data Availability Statement.

The data used to support the findings of this study (including transfection protocols and vector design) are included within the article. All other data used to support the findings of this study (including DNA sequence) are available from the corresponding author upon request.

## 3. Results

### 3.1. Transfer of Transcriptionally Active VL30 Genomes by *γ*-RVV Particles Secreted from the PG13 Packaging Cell Line to Human Embryo Kidney (HEK) 293T Cells.

**Figure 1:**
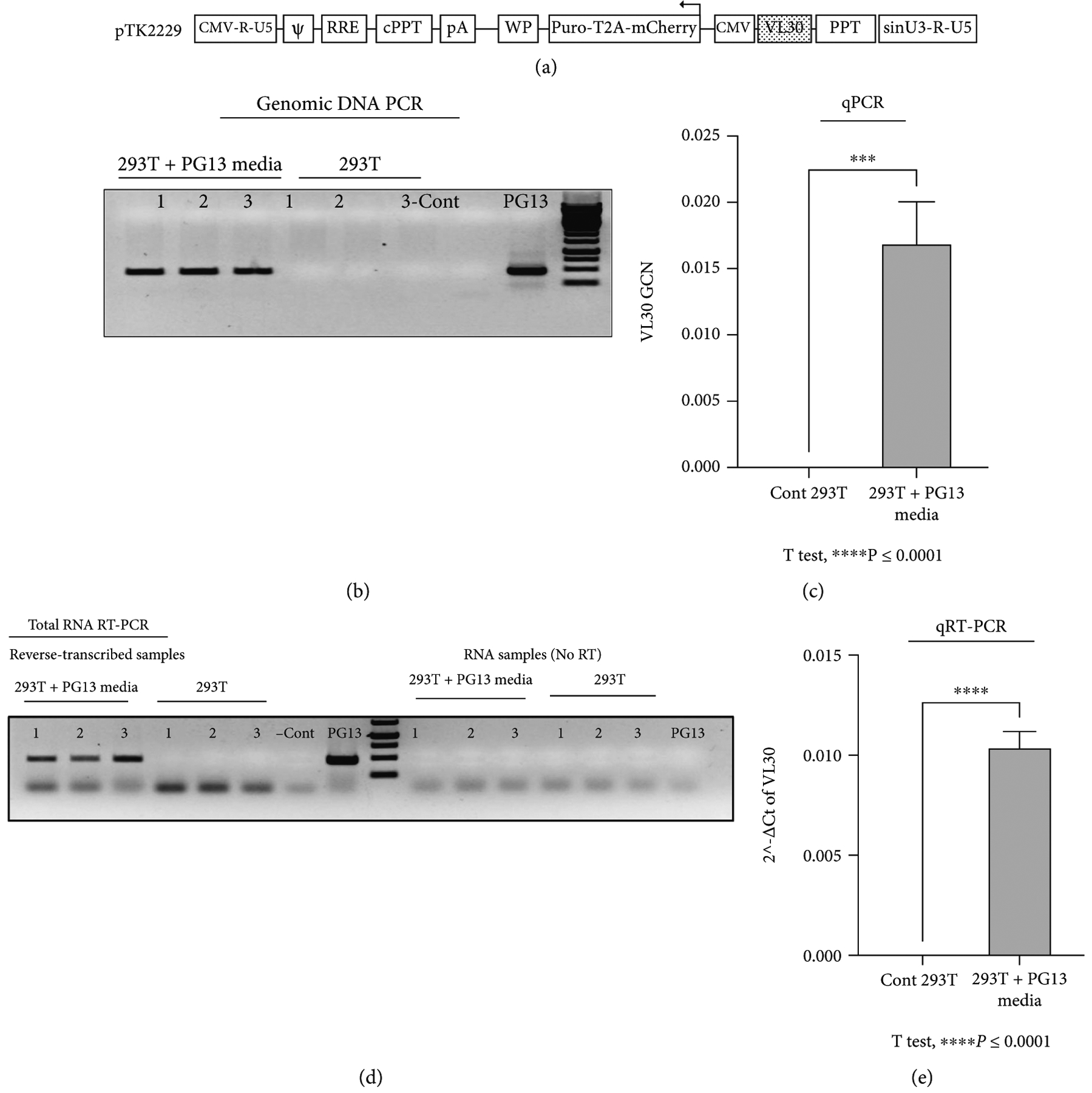
Inadvertently transferred VL30 genomes are transcriptionally active in HEK 293T cells. Detection of VL30 genomes and mRNA in 293T cells following exposure to PG13-conditioned media. (a) Physical map of the lentiviral vector cassette pTK2229 carrying a DNA sequence from the VL30 retrotransposon. The VL30 sequence is shown in a dotted box. It is located downstream of the internal CMV promoter, which is in opposite orientation to the LTRs and thus cannot initiate transcription of the VL30 sequence. The direction of transcription is indicated by an arrow. A CMV promoter replaces the 5′ U3. It is followed by the 5′ R and U5 regions. The packaging signal (Y), Rev response element (RRE), central polypurine tract (cPPT), woodchuck hepatitis virus posttranscriptional regulatory element (WP), internal CMV promoter, and Puro-T2A-mCherry reporter genes are shown. The modified self-inactivating (SIN) 3′ U3 is shown as SINU3. The parental 3′ poly purine tract (PPT) is shown. (b) PCR-based analysis testing the presence of VL30 genomes in 293T cells. DNA was extracted from either naïve or treated (exposed to PG13-conditioned media) 293T cells following 4 passages in culture. The presence of VL30 in the abovementioned DNA samples was determined by PCR. PCRs either in the absence of DNA or with DNA extracted from PG13 cells served as negative and positive controls. PCR products were visualized following gel electrophoresis. (c) Bar graph showing vector copy number analysis of VL30 genomes in 293T cells following exposure to PG13-conditioned media. The qPCR-based assay was done in triplicate. Serial dilutions of DNA from pTK2229 vector-transduced 293T cells served as a standard curve. DNA of naive 293T cells served as a negative control. (d) Employing RT-PCR-based analysis to characterize transcriptional activity of VL30 genomes in 293T cells. Total cellular RNA was extracted from either naïve or treated (exposed to PG13-conditioned media) 293T cells following 4 passages in culture. The presence of VL30 mRNA in the abovementioned RNA samples was determined by RT-PCR. RT-PCR reactions in the absence of RNA or with RNA extracted from PG13 cells served as negative and positive controls. PCR of RNA samples prior to reverse transcription served as controls for DNA contamination. PCR products were visualized following gel electrophoresis. (e) Bar graph showing quantitative (q) RT-PCR analysis of VL30 mRNA in 293T cells following exposure to PG13-conditioned media. The qPCR-based assay was done in triplicate. RNA of naive 293T cells served as a negative control. Levels of the endogenous hACTB mRNA served as an internal reference.

To test the hypothesis that productive VL30 particles released by PG13 packaging cells can infect human cells *in vitro*, the packaging cell line PG13 [6] was purchased from the American Type Culture Collection (ATCC CRL-10686). Conditioned media from PG13 cells was employed on HEK 293T cells. Treated 293T cells were cultured for 2 weeks (4 passages), after which genomic DNA and total mRNA were analyzed to determine the presence of transcriptionally active integrated VL30 genome. As shown in Figures 1(a)–1(e) qPCR and qRT-PCR assays readily detected integrated VL30 genome and mRNA in the abovementioned treated 293T cells. Specifically, up to 1.5 copies of the VL30 genome were detected per 100 293T cells.

### 3.2. PG13 Cells’ Conditioned Media-Mediated Transfer of VL30 Genome to 293T Cells Is Reverse Transcription Dependent.

**Figure 2:**
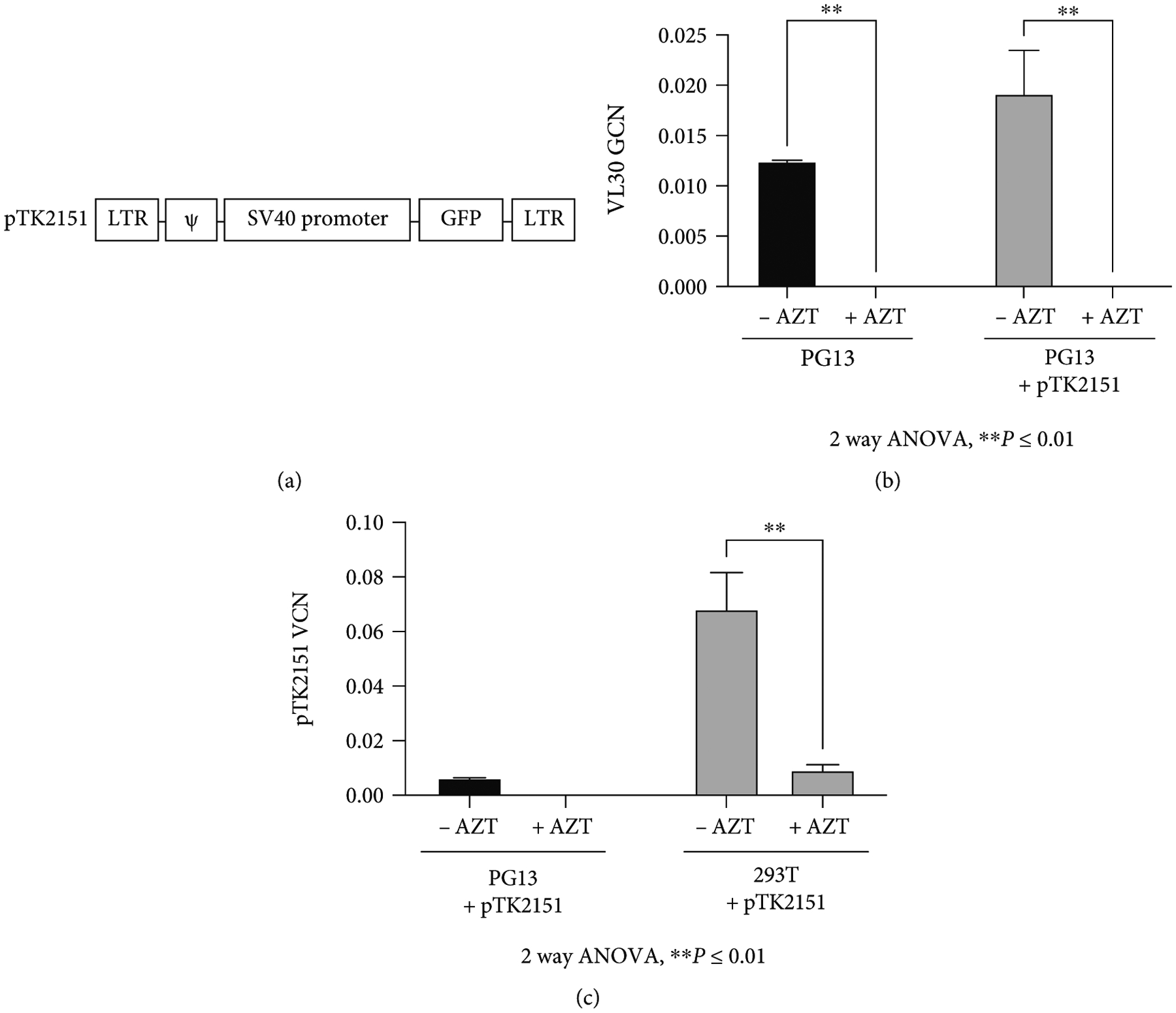
VL30 transduction of 293T cells is reverse transcription dependent. The effects of a reverse-transcriptase inhibitor on VL30 transduction. (a) Physical map of the *γ*-retroviral vector (*γ*-RVV) pTK2151. The 5′ and 3′ non-SIN LTRs are shown. The packaging signal (y), the SV40 promoter, and the GFP reporter gene are shown. (b) Bar graph showing the genome copy number (GCN) of VL30 in 293T cells following exposure to conditioned media collected from either PG13 or PG13 cells transduced with the *γ*-RVV pTK2151. Exposure to the abovementioned conditioned media was done either in the presence or in the absence of the reverse transcriptase inhibitor azidothymidine (AZT 10 *μ*M). The experiment was performed in triplicate. (c) Bar graph showing the vector copy number (VCN) of the *γ*-RVV pTK2151 in 293T cells following exposure to conditioned media collected from either PG13 cells transduced with the *γ*-RVV pTK215 or 293T cells transiently transfected with the pTK2151 vector cassette, a VSV-G envelope, and *γ*-RVV packaging cassettes. Exposure to the abovementioned conditioned media was done either in the presence or in the absence of the reverse transcriptase inhibitor AZT (10 *μ*M). The experiment was performed in triplicate. Significance of the AZT effect on VL30 GCN and pTK2151 VCN was determined by 2-way ANOVA, *P ≤ 0.05 and **P ≤ 0.01.

To support the hypothesis that the transfer of VL30 genomes to 293T cells following exposure to PG13 conditioned media is mediated by *γ*-RVVs, we tested the effects of reverse transcription inhibition on the VL30 genome copy number in the abovementioned treated 293T cells. To this end, PG13 cells were transduced with the *γ*-RVV (pTK2151, Figure 2(a)) from which the green fluorescence protein (GFP) is expressed under the control of a simian virus 40 (SV-40) promoter. Conditioned media was obtained from naïve PG13 cells and from vector-(pTK2151-) transduced PG13 cells and applied onto 293T cells, either in the presence or absence of 10 *μ*M azidothymidine (AZT), a nucleoside reverse transcriptase inhibitor. qPCR-based analysis VL30 genome copy number (GCN) in treated 293T cells demonstrated inhibition of VL30 transduction by AZT (Figures 2(b) and 2(c) and Tables 1 and 2). As expected, the presence of AZT efficiently inhibited the transduction of 293T cells by the pTK2151 *γ*-RVVs, which were generated either in the stable producer cell line PG13 or by transient three-plasmid transduction in 293T cells (Figure 2(c) and Tables 1 and 2).

### 3.3. Increased VL30 Genome Copy Number in 293T Cells following Coculturing with PG13 Cells.

**Figure 3:**
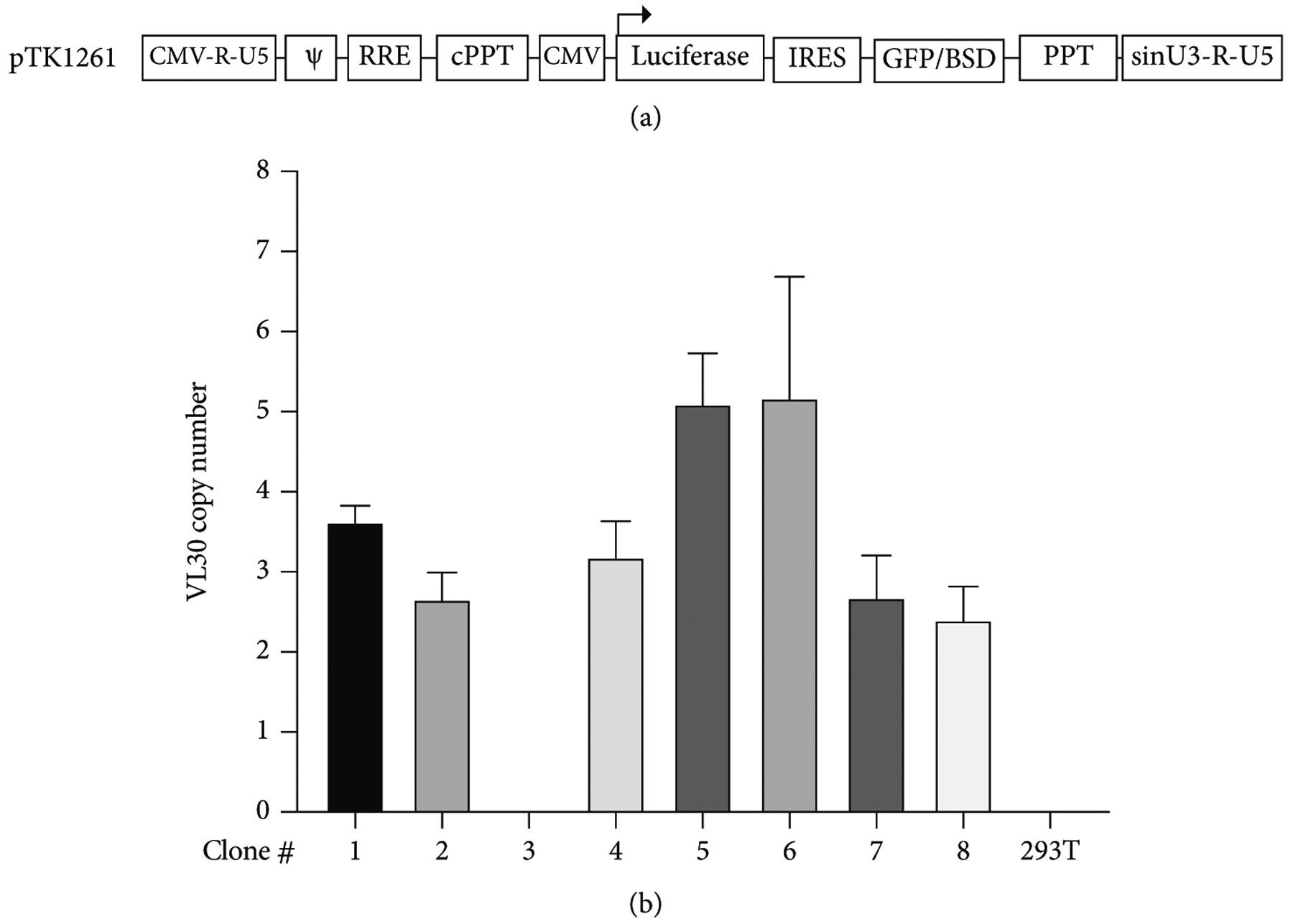
Increased VL30 genome copy number (GCN) in 293T cells, which were cocultured with PG13 cells. (a) Physical map of pTK1261 expressing the firefly luciferase and the GFP-blasticidin selection marker under transcriptional and translational control of a CMV promoter and an internal ribosome entry site (IRES), respectively. (b) Bar graph showing VL30 GCN in single-cell clones of 293T cells. PG13 and pTK2161 vector-transduced 293T cells were cocultured for 7 passages. Single-cell clones of cocultured 293T cells were isolated in the presence of blasticidin (5 *μ*g/ml). GCN in each cell clone was determined by qPCR. pTK2161-transduced 293T served as negative control. The experiment was performed in technical triplicate.

To better characterize VL30 genome function in human cells, we sought to increase VL30 GCN in 293T cells. To this end, we transduced 293T cells with the lentiviral vector pTK1261, which expresses the firefly luciferase and the GFP-blasticidin (BSD) selection marker under the control of the CMV promoter and the encephalomyelitis virus internal ribosome entry site (IRES), respectively. Vector-transduced cells were cocultured with PG13 cells to confluency, at which point PG13 cells were eliminated by blasticidin selection and the vector-transduced cells were recultured with fresh PG13 cells. The coculturing/selection process was repeated 7 times, after which single-cell blasticidin-resistant 293T cell clones were isolated. DNA was isolated from a total of 8 single-cell clones and analyzed by qPCR for VL30 GCN. VL30 genomes were detected in 7 out of 8 single-cell clones. The range of VL30 GCN in the 7 VL30-positive clones was 2.40–5.17 (clones 8 and 6, respectively) VL30 genomes per 293T cell genome (Figures 3(a) and 3(b) and Table 3).

### 3.4. Packaging of an Endogenous Murine Retrotransposon into Productive HIV-1 Particles.

**Figure 4:**
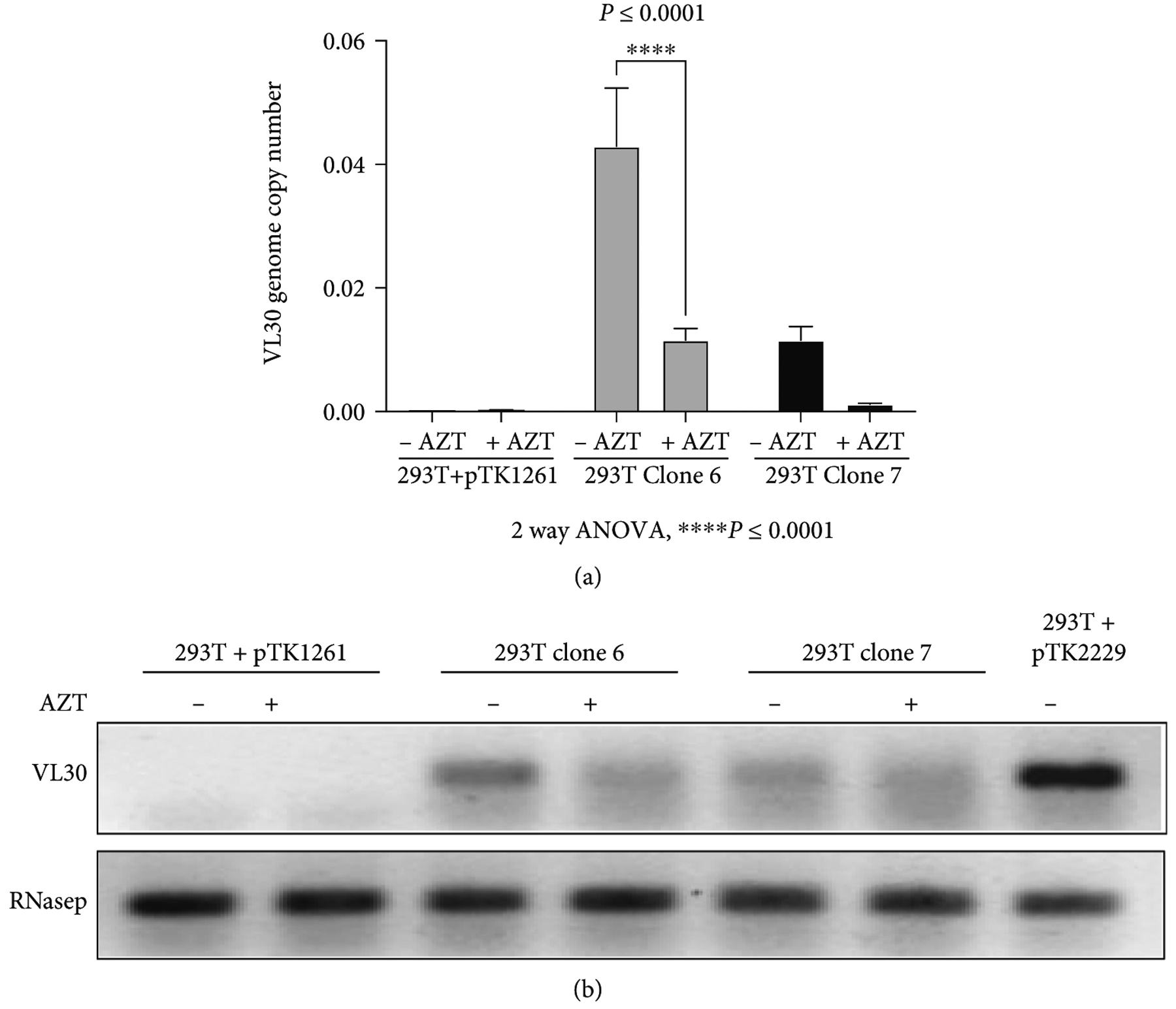
Transfer of VL30 genomes by HIV-1 vector particles. (a) To test the hypothesis that human pathogens, including HIV-1, can potentially transfer VL30 genomes, 293T cell clones 6 and 7 (VL30 GCN of 5.17 and 2.68, respectively) were transiently transfected with the HIV-1 vector packaging and the VSV-G envelope expression cassettes. Vector particles were employed on naïve 293T cells either in the presence or absence of 10 *μ*M AZT. DNA was extracted from treated 293T cells, and the VL30 genome copy number was determined by qPCR. (a) Graph bar showing the VL30 genome copy number in naive 293T cells exposed to lentiviral vector particles generated in cell clones 6 and 7, either in the presence or in the absence of 10 *μ*M AZT. DNA samples extracted from 293T cells comprising the lentiviral vectors pTK1261 and pTK2229 served as negative and positive controls, respectively. The significant reduction in VL30 genome copy number in AZT-treated 293T cells indicates that the observed transfer of VL30 genomes was reverse transcription dependent. PCR amplification products of the endogenous RNaseP gene served as loading controls. *P* values were determined by the 2-way ANOVA test. The experiment was performed in triplicate. (b) Electrophoresis analysis of PCR products following amplification of DNA samples extracted from 293T cells transduced by HIV-1 particles generated in cell clones 6 and 7. DNA samples extracted from 293T cells transduced with the lentiviral vectors pTK1261 and pTK2229 served as negative and positive controls, respectively.

**Figure 5:**
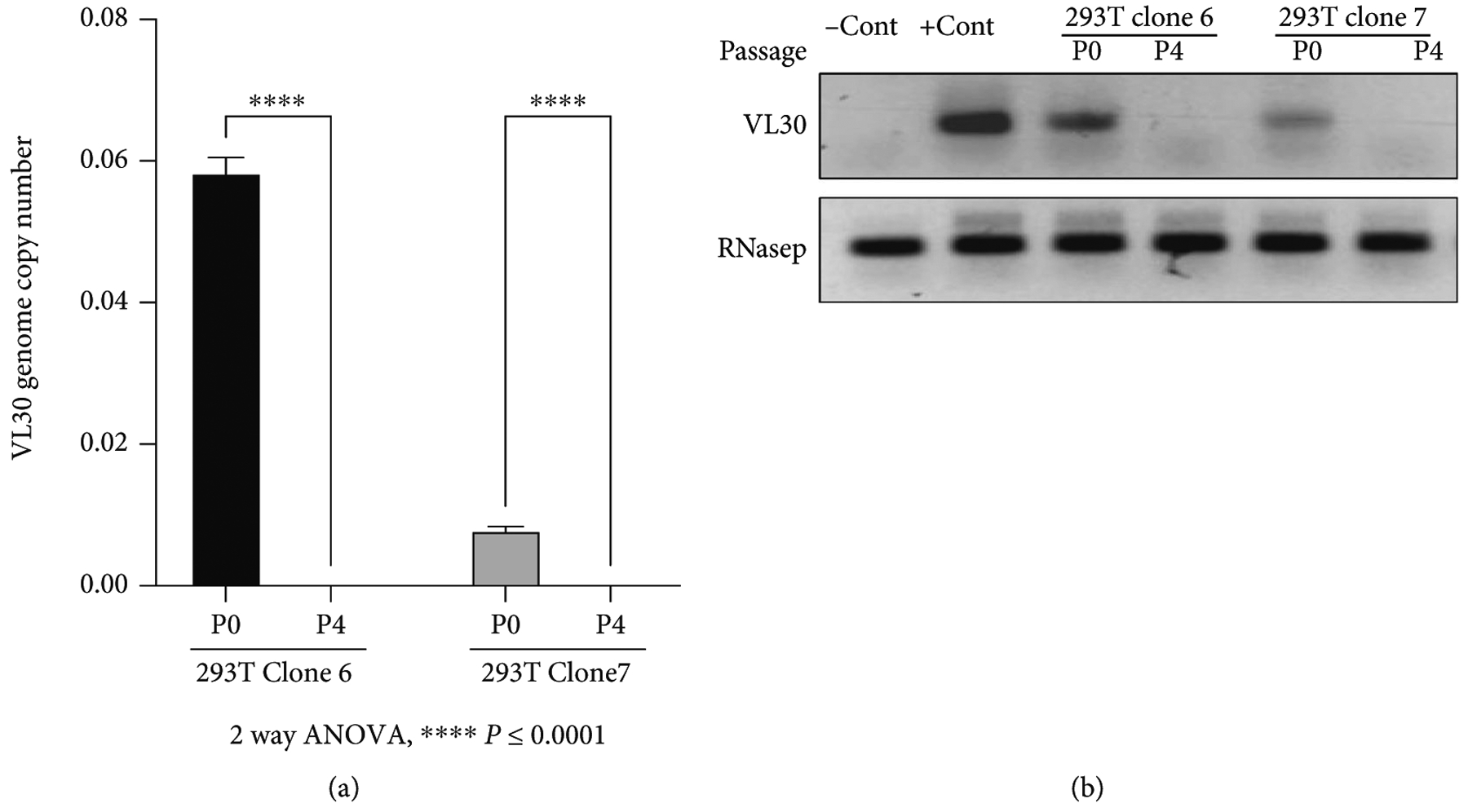
The effect of 293T cell replication on GCN of HIV-1 vector-transferred VL30. (a) Bar graph showing VL30 GCN in 293T cells treated with conditioned media containing HIV-1 vector particles packaged with VL30 genomes. Conditioned media samples were collected from cell clones 6 and 7 following transient transfection with a second-generation HIV-1 vector packaging plasmid and a VSV-G envelope expression cassette. DNA samples of target 293T cells were analyzed by qPCR for the presence of VL30 genomes either at 24 h posttransduction (P0) or following 4 passages (P4). *P* values were determined by the 2-way ANOVA test. The experiment was performed in triplicate. (b) Electrophoresis analysis of PCR products following amplification of DNA samples extracted from 293T cells transduced by HIV-1 particles generated in cell clones 6 and 7. DNA samples extracted from 293T cells transduced with lentiviral vectors pTK1261 and pTK2229 served as negative and positive controls, respectively, and are shown as −Con and +Con, respectively. PCR amplification products of the endogenous RNaseP gene served as loading controls.

To characterize the risk of VL30 genome dissemination within or between human subjects, we sought to evaluate the ability of HIV-1 vector particles to deliver VL30 genomes. To this end, two single-cell clones of 293T cells (clones 6 and 7, which were isolated following the abovementioned coculturing process with PG13 cells) were transiently transfected with a second-generation HIV-1 packaging cassette and a VSV-G envelope expression cassette. Conditioned medium samples from the transiently transfected cells were employed to naïve 293T cells either in the presence or absence of 10 *μ*M AZT. At 24 h after transduction, DNA samples were extracted and analyzed by qPCR to determine the presence of VL30 genome. As shown in Figures 4(a) and 4(b) and Table 4, we readily detected VL30 genomes in 293T cells that were exposed to conditioned media of clones 6 and 7 in the absence of AZT. However, a significant reduction in the VL30 genome copy number was observed in 293T cells that were exposed to the abovementioned conditioned media in the presence of AZT (an inhibitor of HIV-1 reverse transcriptase). These data suggest that HIV-1 particles can mediate the transfer of VL30 genomes between human cells. Furthermore, as shown in Tables 4 and 3, the level of VL30 GCN in 293T cells treated with conditioned media from clones 6 and 7 (4.29 and 1.15 genome copies per 100 cells, respectively) correlated with the number of VL30 genomes in these cell clones (5.17 and 2.68 genomes per cell, respectively). To characterize the ability of HIV-1 reverse-transcribed VL30 genomes to integrate into human cell chromatin, we compared the VL30 genome copy numbers observed at 24 h (P0) and at 4 passages (P4) after transduction. As shown in Figures 5 (a) and 5(b) and Table 5, in contrast to the *γ*-retroviral integrase, the HIV-1 integrase failed to integrate reverse-transcribed VL30 genome into the chromatin of human 293T cells. This phenomenon could be explained by the lack of compatibility between the attachment sites (*att sequences*) of the VL30 LTRs and the HIV-1 integrase [34] and is in line with an earlier report describing the packaging of chimeric HIV-1/*γ*-RVV genomes by HIV-1 vector particles [35].

### 3.5. Clinical-Grade *γ*-RVVs Produced in the PG13 Packaging Cell Line Inadvertently Transfer VL30 Genomes to CAR-T Cells.

**Figure 6:**
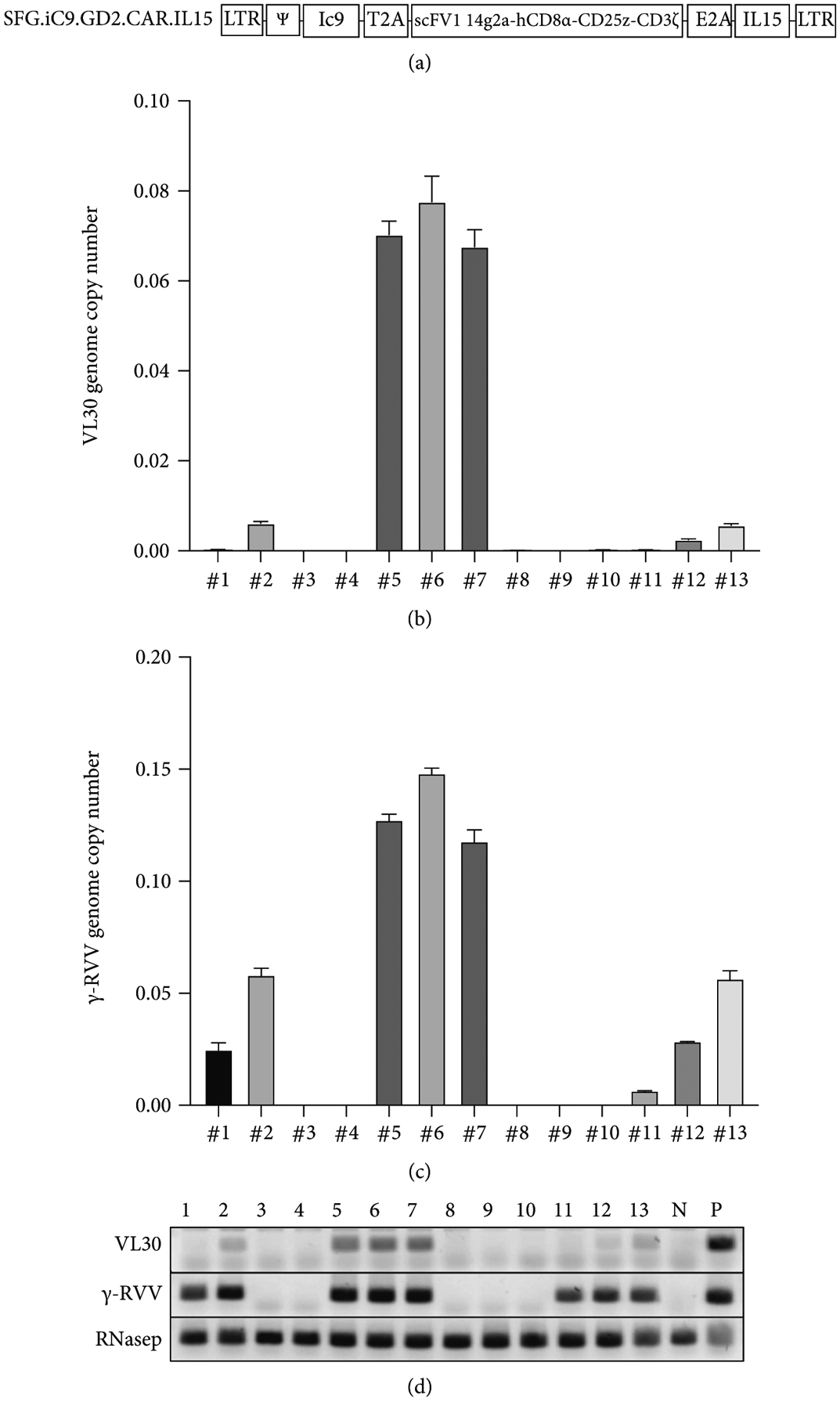

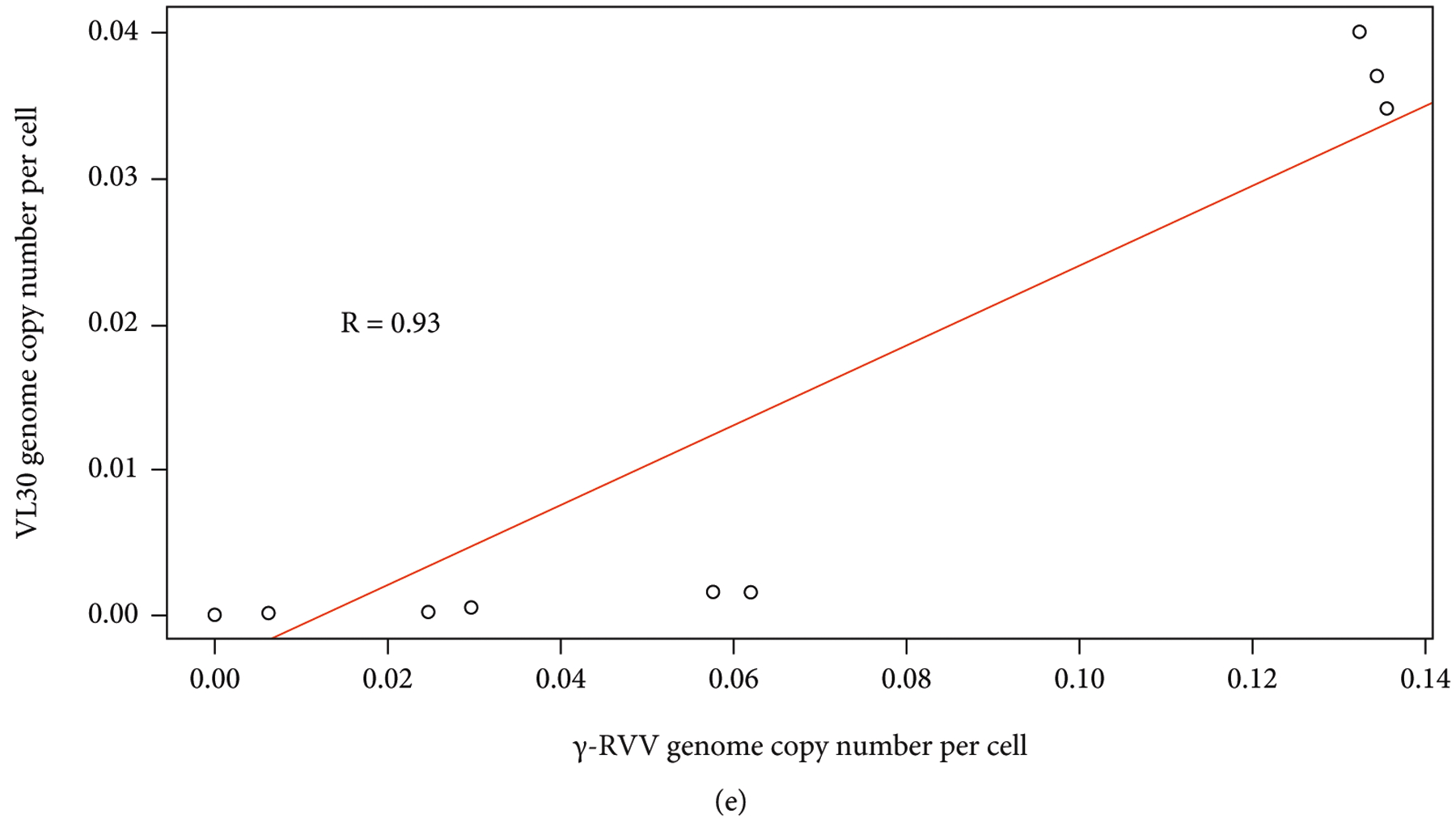
qPCR-based analysis of VL30 GCN and *γ*-RVV VCN in primary human T-cells. Naïve primary human T-cells were treated with the chimeric antigen receptor- (CAR-) carrying *γ*-RVV SFG.iC9.GD2.CAR.IL15 (samples 1, 2, 5, 6, 7, 11, 12, and 13). The vector was produced in the stable packaging cell line PG13. Samples 3, 4, 8, 9, and 10 were not exposed to the abovementioned CAR-carrying *γ*-RVV and served as biologic negative controls. DNA samples extracted from the abovementioned controls and vector-treated cells were used to determine GCN and VCN of the murine endogenous retrotransposon VL30 and the *γ*-RVV vector, respectively. DNA from naïve 293T cells served as a technical negative control. DNA from 293T cells transduced with the lentiviral vectors pTK2229 and pTK2151 served as positive controls for VL30 and *γ*-RVV genome amplification, respectively. Amplification of the endogenous human gene hRNaseP served as a loading control. The assay was performed in technical triplicate. (a) Physical map depicting the structure of the CAR-carrying *γ*-RVV, SFG.iC9.GD2.CAR.IL15. The vector’s non-self-inactivating (non-SIN) long-terminal repeats (LTRs) and the packaging signal y are shown. The inducible caspase-9 suicide gene, Ic9, is shown. The self-cleavable peptide from the thosea asigna virus (T2A) and the equine rhinitis virus (E2A) is shown. Sequences encoding the CAR including the single-chain variable fragment (scFv1) directed to the NB-antigen GD2 of the disialoganglioside GD2 (14g2a), the CD8a stalk and transmembrane domain, the CD28 intracellular domain, and the CD3z chain are shown. The human IL15 cDNA containing sequence is shown. (b) Graph bar showing GCN of VL30 in the abovementioned primary human T-cells. The experiment was performed in technical triplicate. (c) Graph bar showing VCN of the *γ*-RVV SFG.iC9.GD2.CAR.IL15 in the abovementioned primary human T-cells. The experiment was performed in technical triplicate. (d) Gel electrophoresis analysis of DNA amplification products of VL30, *γ*-RVV, and hRNaseP sequences generated in the course of the abovementtioned gPCR assay. DNA of naïve 293T cells (N) served as a technical negative control for amplification of VL30 and *γ*-RVV sequences. DNA from 293T cells transduced with the lentiviral vectors pTK2229 and pTK2151 served as positive controls (P) for VL30 and *γ*-RVV genome amplification, respectively. (e) Significant correlation between VL30 and *γ*-RVV genome copy number in human T-cells transduced with CAR-expressing *γ*-RVV. Linear regression graph demonstrating the relation between VL30 and *γ*-RVV genome copy number. The R value is indicated.

Next, we sought to evaluate the risk of inadvertent transfer of VL30 retrotransposons to human cells in a relevant clinical setting. To this end, we determined the presence of VL30 genomes in 8 populations of clinical-grade CAR-T cells and in 5 populations of naïve primary human T-cells. All cell populations were obtained from healthy human donors. CAR-T cells were generated by a clinical-grade transduction protocol using *γ*-RVV generated in PG13 producer cell lines, as described in [29]. A total of 13 samples of primary human cells were analyzed by qPCR for the presence of VL30 and *γ*-RVV genomes. DNA samples 3, 4, 8, 9, and 10 were extracted from control human T-cells that were not treated with the abovementioned *γ*-RVV (employed as negative control). As shown in Figures 6(a)–6(d) and Table 6, *γ*-RVV genomes were detected in all 8 populations of *γ*-RVV-treated T-cells. The vector copy number (VCN) of *γ*-RVV ranged from ~13.5 to ~0.6 vector copies per 100T-cells (in samples 6 and 11, respectively). VL30 genomes were detected in 6 out of 8 T-cell populations exposed to *γ*-RVV (cell populations 2, 5, 6, 7, 12, and 13). In these T-cell populations, VL30 GCN ranged from ~40 to ~0.5 genome copies per 1000 cells (in samples 5 and 12, respectively). Two populations of *γ*-RVV-treated T-cells (samples 1 and 11) were observed to be negative for the presence of VL30 genome. Importantly, there was a correlation between *γ*-RVV VCN and VL30 GCN in the different T-cell populations (Figure 6(e)). This observation raises the possibility that the low levels of VL30 genomes in samples 1 and 11, which exhibited the lowest levels of *γ*-RVV VCN, were below the qPCR detection level employed in this study.

### 3.6. VL30 Genomes Are Transcriptionally Active in Primary Human T-Cells.

**Figure 7:**
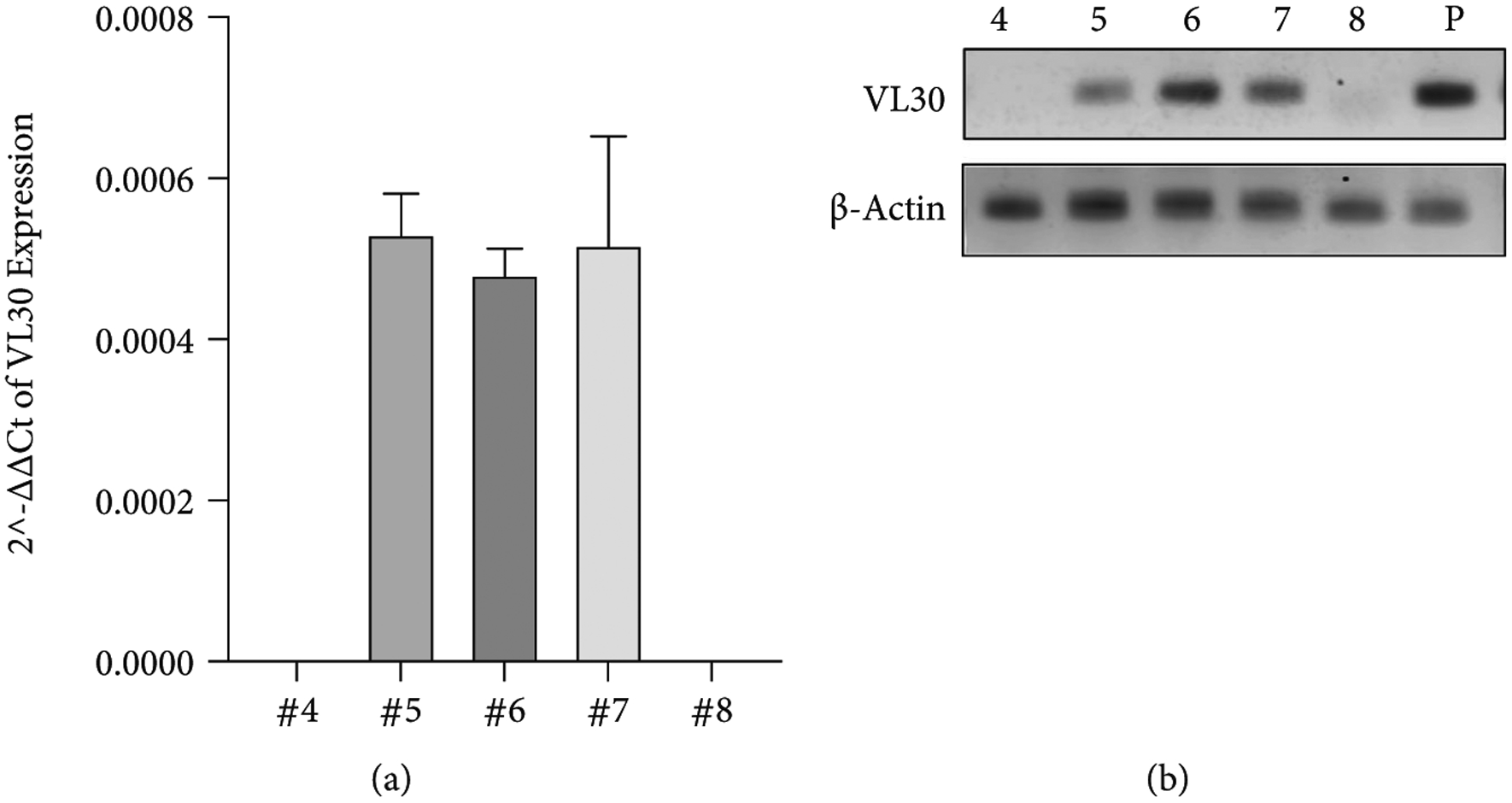
Quantification of VL30 expression in human T-cells. (a) Bar graph showing VL30 expression relative to the endogenous *β*-actin gene. The qRT-PCR was performed in technical triplicate on RNA samples extracted from samples 4–8. Note that samples 4 and 8, in which VL30 genomes could not be detected (Figure 6), served as negative controls. (b) Gel electrophoresis analysis showing the DNA products of the abovementioned qRT analysis. Amplification products of RNA samples extracted from 293T cells transduced with the lentiviral vector pTK2229 (Figure 1) served as a positive control (P). Amplification products on of the *β*-actin mRNA are shown.

Aware of the oncogenic potential of VL30 mRNA, we investigated the transcriptional activity of VL30 genomes in the abovementioned VL30-containing human T-cell populations. To this end, we employed the reverse transcriptase (RT) qPCR assay on RNA samples extracted from T-cell populations 5, 6, and 7, as well as from populations 4 and 8, which were not treated with *γ*-RVVs and served as negative controls. As shown in Figures 7(a) and 7 (b) and Table 7, VL30 transcripts were detected in all RNA samples from T-cell populations 5–7. These data suggest that VL30 genomes that were inadvertently transferred to primary human T-cells during *γ*-RVV transduction were transcriptionally active.

### 3.7. Short Sequences of Homology between the Human Genome and Various VL30 Genomes.

The transcriptional activity of VL30 genomes in primary human T-cells and the ability of HIV-1 vector particles to mobilize VL30 genomes from human cells (as shown earlier in Figures 4 (a) and 4(b) and Table 4) increase the potential for recombination between VL30 genomes and either human or/and HIV-1 genomes [28, 36–38]. Prompted by this possibility, we searched for sequence homology between various VL30 genomes and either the HIV-1 genome or the human genome. We could not detect sequence homology between VL30 genome and the HIV-1 genome. Importantly, multiple short sequences of ~30 bp in VL30 genomes were determined to be homologous to sequences throughout the human genome. Most of the abovementioned VL30-homologous human sequences were located in interspersed repeats, including LTRs, LINEs, and SINEs, or simple repeats (Table 8).

## 4. Discussion

Following successful clinical trials, in 2017 [39, 40], the US Food and Drug Administration (FDA) approved Tisagenlecleucel (Kymriah^®^ Novartis) and axicabtagene ciloleucel (Yescarta^®^ Kite Pharma) as the first two gene therapy-based immunotherapy drugs for patients with hematologic malignancies. The active component of the novel anticancer drugs is made of autologous T-cells that express viral vector-delivered chimeric antigen receptors (CARs). The CAR-Ts in both drugs target the B cell CD19 protein. Initially, Kymriah^®^ and Yescarta^®^ were indicated for young (under 25 years old) patients with acute lymphocytic leukemia (ALL) or adult patients with large B-cell lymphoma, respectively, who relapsed or did not respond to two conventional anticancer treatments [39, 40]. HIV-1-based vectors generated by transient four-plasmid transfection of human cells deliver the CAR expression cassette to Kymriah^®^ CAR-Ts. Generation of Yescarta^®^ CAR-Ts is mediated by transduction with the *γ*-RVV PG13-CD19-H3. The vector is produced in a stable producer cell line, which was isolated by Kochenderfer et al. [5] as a single-cell clone (clone H3) following transduction of murine PG13 packaging cells [6] (ATCC CRL-10686). Prior to and since 2017, the PG13 packaging cell line and its derivatives have been employed to generate *γ*-RVV-transduced T-cells for various anticancer protocols [41–50]. Importantly, all tested *γ*-RVVs employed in the abovementioned clinical trials were determined to be free of replication-competent retroviruses (RCRs) [42, 44]. In 2020, the FDA and the European Medicines Agency (EMA) approved a second medicine premised on *γ*-RVV-transduced CAR-T cells, Tecartus, as an immunotherapy medicine [51, 52]. Tecartus is indicated for the treatment of adult patients with relapsed/refractory mantle cell lymphoma (MCL). The *γ*-RVVs employed to generate Tecratus and Yescarta^®^ CAR-T cells are identical and are generated in the same PG13 producer cell line. Although stable packaging cell lines facilitate the production of RVVs [7], the transcriptional activity and replication of endogenous murine retrotransposons [8–10, 53] raise biosafety concerns regarding copackaging of endogenous murine retrotransposons along with clinical-grade *γ*-RVVs. Indeed, earlier preclinical studies reported on efficient copackaging of the murine VL30 retrotransposon with *γ*-RVVs generated in various murine stable packaging cells [11–15, 17]. VL30 is a nonautonomous LTR-retrotransposon. Markopoulos et al. [54] identified 372 VL30 sequences in the mouse genome. The murine VL30 sequences include 86 full-length genomes comprising 2 LTRs and all *cis* elements required for retroviral replication, including a primer-binding site (PBS), a packaging signal, and a polypurine tract (PPT). VL30 promoter/enhancer sequences in the 5′ LTR contain multiple transcription factor binding sites, which regulate VL30 mRNA expression and potentially exert transcriptional *cis* effects on neighboring host genes [53–55]. VL30 genomes do not encode functional proteins [19]. Thus, the entire VL30 life cycle depends on either exogenous or endogenous retroviral proteins [19, 54–56]. However, VL30 mRNAs function as lncRNAs, most of which contain two RNA motifs, which efficiently bind and alter the interaction of the human polypyrimidine tract-binding protein-associated splicing factor (PSF) with its natural DNA and RNA target sites [20–22]. PSF belongs to the Drosophila behavior/human splicing (DBHS) protein family and interacts with nuclear and cytoplasmic proteins, as well as with RNA and DNA target sequences. Nucleic acid/PSF interactions are mediated by a DNA-binding domain (DBD) and two RNA-binding domains in the PSF protein [26, 57]. PSF is a multifunctional protein involved in major physiological and pathological pathways, including oncogenesis, DNA repair, RNA processing, cytokine release, viral infection, and neurodegeneration [20, 26, 57–60]. In mammalian cells, PSF is one of three proteins whose association with the scaffold lncRNA NEAT1_2 initiates a liquid-liquid phase separation process and the formation of membraneless nuclear paraspeckles organelles, which regulate gene expression via several mechanisms, including sequestration of nucleoplasmic proteins and RNA molecules. An increase in the paraspeckle number or size secondary to overexpression of the NEAT1_2 mRNA further recruits paraspeckle proteins, diminishes their nucleoplasmic concentration, and alters their ability to regulate the expression of genes involved in major physiological pathways [61, 62]. VL30 and NEAT1_2 mRNAs are not the only RNA molecules with which PSF interacts. A study by Li et al. demonstrated PSF binding to four human mRNAs, which are overexpressed in cancer cells [20]. The ability of PSF/VL30 mRNA interaction to promote metastasis of human melanoma cells in immunodeficient mice indicated the oncogenic potential of VL30 and other PSF-binding ncRNAs. Garen and Song outlined a molecular model of ncRNA/tumor suppressor protein (TSP) complex-induced tumorigenesis [63, 64]. In this model, PSF-like TSPs bind to and inhibit the transcription of protooncogenes. Binding of PSF to lncRNAs, such as VL30, results in PSF dissociation from its genomic target sequence and consequent activation of transcriptionally suppressed protooncogenes. However, various experimental systems demonstrated that the direction and mechanisms by which PSF/ncRNA complexes affect oncogenic pathways are cell type dependent [65–69]. Similarly, notwithstanding the role of NEAT1 and PSF in inflammation and activation of the innate immune response [24, 70–73], the paraspeckles’ effects on viral infection are pathogen-specific [24, 61, 68, 74–79]. Based on the multiple mechanisms by which PSF mediates its functions, it is difficult to predict the effects of VL30 mRNA expression on inflammatory (e.g., cytokine release) or oncogenic pathways in human T-cells. However, the oncogenic potential of PSF-binding mRNAs was considered in the wake of an earlier gene therapy preclinical study demonstrating the presence of VL30 genome in lymphoma cells following bone marrow transplantation of *γ*-RVV-transduced simian hematopoietic stem cells [16]. In this study, the abovementioned VL30 genome containing lymphoma cells did not express VL30 mRNAs, which suggested that additional mechanisms of VL30-mediated insertional mutagenesis can contribute to the oncogenic potential of VL30 genome. Reverse-transcribed VL30 genomes preferentially integrate in proximity to transcriptional start sites, and their distribution among mouse chromosomes is not random [54]. VL30-mediated insertional mutagenesis can alter host gene expression via various mechanisms, including (a) directly disrupting of host regulatory and protein-encoding sequences, (b) transcriptionally activating of neighboring host genes via transcription factor-binding sites in the VL30 LTRs [54, 55, 80–82], and (c) spreading host-mediated epigenetic silencing from the VL30 LTR to neighboring genes [83–85]. The integration of transcriptionally active VL30 genomes into patients’ chromatin raises an additional biosafety concern regarding the possibility of emerging novel retroviruses following recombination between VL30 genomes and either endogenous or exogenous retroviruses. Genomic analysis of the transforming and replication-defective Kirsten and Harvey murine sarcoma viruses (Ki-MSV and Ha-MSV, respectively) hints at the recombinational potential of VL30. The genomes of these viruses comprise sequences from three sources: the rat VL30, the Moloney murine leukemia virus LTRs, and the Kirsten and Harvey viral *ras* genes [37, 38, 86]. In a different study, Itin and Keshet identified DNA recombinants comprising the VL30 LTRs and sequences with homology to the murine leukemia virus (MuLV) *gag* and *pol* genes [28]. Several VL30-specific factors potentially contribute to the risk of emerging novel VL30-based recombinants; these factors include (a) the presence of multiple short sequences in the human genome that exhibit high identity to sequences in VL30 genomes and (b) packaging of VL30 genomes into productive human retroviral particles. Under natural conditions, the sequence and structural differences between *cis*-regulatory elements involved in all steps of the human retroviral (e.g., HIV-1 and HTLV) and VL30 life cycle minimize the risk of horizontal transfer of murine VL30 genomes by HIV-1 particles. For instance, there is no homology between the PBS sequences of VL30 strains (which are mostly complementary to the tRNA^Gly^) and the sequence of HIV-1 PBS (which is complementary to the tRNA^Lys^). Similarly, the 3′ polypurine tract (PPT) of VL30 shows no sequence homology with that of HIV-1 [18, 19, 53, 54, 87]. Furthermore, there is no sequence homology between VL30 and HIV-1 packaging signals. Notwithstanding the lack of sequence homology between key VL30 and HIV-1 *cis* elements, in this study, for the first time, we showed reverse transcription-dependent delivery of transcriptionally active VL30 genomes to naïve human cells by HIV-1 vector particles. However, the loss of HIV-1 vector-delivered VL30 genomes in replicating cells (following 4 passages in culture) suggested that reverse-transcribed VL30 DNA failed to integrate into the transduced cells’ chromatin. This phenomenon can be attributed to the incompatibility between the VL30 *att* sites and the HIV-1 integrase [34] or/and to incompatibility between the VL30 polypurine tract (PPT) and the HIV-1 reverse transcriptase, which may alter the last step of reverse transcription and consequently leads to the formation of mostly episomal single-LTR circles (which, unlike fully reverse-transcribed linear double-stranded genomes, cannot serve as an integration template) [88]. This notion is supported by an earlier study demonstrating the delivery of productive *γ*-retroviral vectors by HIV-1 particles, which, similar to the abovementioned VL30 genomes, failed to integrate. In this study, genomic analysis of *γ*-retroviral vector genomes (following delivery via HIV-1 vector particles) demonstrated the presence of episomal single-LTR circles and no linear vector forms [35]. Importantly, incorporation of HIV sequences comprising the rev-response element (RRE) to *γ*-retroviral vector genomes significantly increased their titers following packaging by HIV-1 particles. Premised on these findings, as well as the natural reversion rate of mutated HIV-1 genomes [89, 90] and the VL30 recombinational potential [28, 37, 38], it is possible to speculate that in the presence of active HIV-1 genomes, novel VL30 recombinants can evolve to maximize their transfer by HIV-1 particles. This theoretical scenario raises biosafety concerns regarding the mobilization of VL30 genomes within CAR-T-treated patients and in the worst scenario within the treated patients’ community. Additional consideration should be given to the fact that PFS-mediated alternative splicing is central to T-cell activation. Thus, there is a possibility that CAR-T cell activation (via the CAR CD28-costimulatory domain) may be altered following binding/sequestration of nuclear PFS by VL30 mRNA [91–93].

Notwithstanding these biosafety concerns, more than a thousand patients with incurable oncologic diseases have been successfully treated with CAR-T cells [94, 95]. To date, not a single clinical report has described proliferative abnormalities that could be attributed to the inadvertent contamination of therapeutic T-cells with VL30 genomes [42, 44, 95]. Theoretically, it is remotely possible that in contrast to the PG13 producer cells characterized in this study, specific vector producer cell clones that were individually isolated for specific clinical applications did not secrete VL30 genome comprising *γ*-retroviral particles. Furthermore, this study did not determine the presence of VL30 genome in patient-administered CAR-T cells. Thus, the half-life of VL30 genome-containing T-cells as well as the level of VL30 mRNA expression and its effects on CAR-T function have not been evaluated *in vivo*. These unknown variables and the clinical history of a large number of CAR-T-treated patients suggest that there are no imminent biosafety risks associated with VL30 genome-containing CAR-T cells. Importantly, short- and long-term biosafety concerns associated with engineering CAR-Ts by murine packaging cell line-derived *γ*-retroviral vectors could be avoided by using human packaging cell lines to generate the same CAR-carrying *γ*-retroviral vectors. Importantly, this study underscores the importance of addressing the potential biosafety risks associated with the transfer of nonhuman endogenous retroviruses and potentially nonautonomous retrotransposons to patients undergoing novel therapeutic procedures. [96–100]

## 5. Conclusions

*γ*-RRV particles produced by the stable PG13 packaging cell line deliver transcriptionally active VL30 genomes to human cells. HIV-1 vector particles package and deliver VL30 genomes to naive human cells in culture. Transcriptionally active VL30 genomes were detected in clinical-grade CAR-Ts generated by a clinical grade protocol. The findings of this study raise biosafety concerns regarding the use of murine packaging cell lines in the past and in ongoing clinical applications.

## Figures and Tables

**Table 1:** The effects of a reverse transcriptase inhibitor of VL30 transduction. The raw data described in Figure 2(a) is presented. The conditioned media producing cell lines are outlined. The absence and presence of AZT (10 *μ*M) at the time of exposure to the abovementioned conditioned media are indicated by − and + signs, respectively. The copy number of VL30 genomes (GCN) in the abovementioned 293Ts is indicated. ND indicates VCN levels that were lower than the lower detection level by qPCR. The values of standard deviation (std) are shown.

Producing cell	AZT	VL30 GCN	Std
PG13	−	0.0124	0.0011
	+	ND	
PG13 + pTK2151	−	0.0168	0.0068
	+	ND	

**Table 2:** The effects of a reverse transcriptase inhibitor of VL30 transduction. The raw data described in Figure 2(b) is presented. The conditioned media producing cell lines are outlined. The absence and presence of AZT (10 *μ*M) at the time of exposure to the abovementioned conditioned media are indicated by − and + signs, respectively. VL30 GCN in the abovementioned 293Ts is indicated. ND indicates VCN levels that were lower than the detection level obtained through qPCR. The values of standard deviation (std) are shown.

Producing cell	AZT	*γ*-RVV VCN	Std
293T + pTK2151	−	0.0682	0.0114
	+	0.0092	0.0016
PG13 + pTK2151	_−_	0.0064	0.0002
	+	ND	

**Table 3:** The table outlines the raw data of the experiments described in Figure 3(b). Clone numbers, values of VL30 GCN, and standard deviations (std) are shown.

Clone #	VL30 GCN	Std
1	3.62	0.21
2	2.66	0.34
3	ND	
4	3.18	0.45
5	5.10	0.63
6	5.17	1.51
7	2.68	0.52
8	2.40	0.42
293T	ND	
DW	ND	

**Table 4:** Data of the qPCR assays described in Figure 4(a). The vector and the conditioned media employed on the target 293T cells are indicated. The presence or absence of 10 *μ*M AZT at the time of transduction is indicated. DNA samples extracted from 293T cells transduced with the lentiviral vector pTK1261 served as negative controls. VL30 genome copy number per cell (GCN) in conditioned media-treated 293T cells and standard deviations (std) are shown. PCR amplification products of the endogenous RNaseP gene served as loading controls. *P* values were determined by the 2-way ANOVA test. The experiment was performed in triplicate.

Cell line	AZT	VL30 GCN	Std
293T + pTK1261	−	Not detected	
	+	Not detected	
293T+	−	0.0429	0.0094
Clone 6 conditioned media	+	0.0116	0.0018
293T+	−	0.0115	0.0022
Clone 7 conditioned media	+	0.0012	0.0002

**Table 5:** The table outlines the data employed in Figure 5(a). HIV-1 vector particles were generated in 293T cell clones 6 and 7 and employed on naive 293T cells. DNA was extracted from treated cells at either 24 h (P0) or following 4 passages (P4) after treatment and analyzed by qPCR for the VL30 genome copy number. The experiment was done in triplicate. The target cell line (293T cells) and the cell lines in which the HIV-1 particles containing conditioned media were generated are shown. The number of target cell passages (P0 and P4) is shown. Values of the VL30 genome copy number per target cell and the standard deviation (std) are shown.

Cell line	Passage	VL30 GCN	Std
293T+	P0	0.0583	0.0022
Clone 6 conditioned media	P4	Not detected	
293T+	P0	0.0078	0.0022
Clone 7 conditioned media	P4	Not detected	

**Table 6:** Genome copy number (GCN) and vector copy number (VCN) of VL30 and *γ*-RVV (respectively), in DNA samples extracted from primary human T-cells as determined by qPCR analysis. DNA samples 1, 2, 5, 6, 7, 11, 12, and 13 were extracted from human T-cells treated with the *γ*-RVV SFG.iC9.GD2.CAR. IL15. DNA of samples 3, 4, 8, 9, and 10 extracted from naïve primary human T-cells and served as biological negative controls. DNA extracted from naïve 293T cells served as technical negative control (−cont). ND indicates that the level of targeted sequences (VL30 and *γ*-RVV) in the tested DNA sample was under the level of detection.

Sample #	VL30		*γ*-Retroviral vector	
	VCN	Std	VCN	Std
#1	0.0002	0.0001	0.0249	0.0030
#2	0.0061	0.0004	0.0582	0.0029
#3	ND		ND	
#4	ND		ND	
#5	0.0703	0.0030	0.1274	0.0025
#6	0.0776	0.0057	0.1482	0.0023
#7	0.0676	0.0038	0.1178	0.0051
#8	0.0000	0.0000	ND	
#9	ND		ND	
#10	0.0001	0.0001	ND	
#11	0.0001	0.0001	0.0066	0.0001
#12	0.0025	0.0001	0.0286	0.0001
#13	0.0057	0.0004	0.0565	0.0036
−Cont	ND		ND	

**Table 7:** The table outlines the raw data described in Figure 7. Values of 2^*DCt* and standard deviation (std) are shown. ND indicates mRNA levels below the level of detection. *P* indicates positive control (RNA extracted from 293T cells transduced with the lentiviral vector pTK2229).

Sample #	2^*ΔCt*	Std
#4	ND	
#5	0.00053	4.98 × 10^−5^
#6	0.00048	3.10 × 10^−5^
#7	0.00052	1.34 × 10^−4^
#8	ND	
*P*	0.03200	1.81 × 10^−3^

**Table 8:** Sequence homologies between various VL30 genomes and the human genome. The Genome Browser of the University of California Santa Cruz Genomic Institute (https://genome.ucsc.edu/) was employed to identify sequence homologies between various VL30 genomes and the human genome. The accession numbers associated with the sequence of the various VL30 genomes, which were employed in the VL30/human sequence homology screen, are indicated. The positions of the first and last (start and end, respectively) of VL30 nucleotides in the homologous sequence are shown. VL30 nucleotides that matched their respective nucleotides in homologous human sequences are shown in capital letters. The chromosome number of human chromosomes containing sequences with homology to VL30 genome sequences is indicated. The orientations of the relevant homologous strands are shown. The positions of the first and last (start and end, respectively) nucleotides in the human chromosomes with homology to the relevant VL30 sequence are shown. The repetitive sequences in the human chromosomes with homology to the abovementioned VL30 genomes are defined.

VL30				Chromosome location				
Accession number	Start	End	VL30 sequence	Chromosome	Strand	Start	End	Repeats
	3692	3844	GATAATGGtCCTGCCTT TgttgCccaggTAagTCAGggtGTggccAAGtatTTagAggTcaAAtaaAAatTCCATT GTGtgTAcaGACCtCAGAGCTC aGaaaAGaTAaAAaAgAatAAatAaAaCtctAAAcAgAcctTgACAAAATTAATC	Chromosome 1	+	90033669	90033696	LINE
				Chromosome 2	+	112924425	113176587	LINE
					+	38868787	38868812	MER5A hAT-Charlie
					−	226901221	226901241	LINE
				Chromosome 3	+	160777702	160777732	LINE
					+	11580350	11580370	LINE
AF486451.1				Chromosome 4	+	167811047	167811066	LINE
				Chromosome 6	+	128820061	128820085	LINE
				Chromosome 7	+	36731671	36731699	LINE
				Chromosome 14	+	32417785	32417804	
					−	76801742	76801771	LINE
				Chromosome 15	+	54047796	54047817	
	1347	1375	CTGTGTGtGTCT.TGTGTGTCTCTT.TGTGT	Chromosome 1	−	5744701	5744788	SIMPLE
				Chromosome 4	−	8027613	8027633	
				Chromosome 5	−	117789902	117790357	SINE
					+	38168444	38168464	LINE
M21123.1				Chromosome 9	+	1592611	1592632	
				Chromosome 12		12048235	12048332	
				Chromosome 18	+	47325316	47325542	LINE
					+	79829326	79829346	SIMPLE
				Chromosome 19	−	42595166	42595192	SIMPLE
	1938	1979	GAAAAACGGTTTctGTgTgtgTcttTTtTgTCTCTTTGTGTT	Chromosome 4	+	121936835	121936854	LTR
				Chromosome 6	−	78148383	78148408	SINE/LINE
				Chromosome 10	+	47191948	47191976	SIMPLE
				Chromosome 11	+	66327659	66327696	
	1632	1676	TGTCTgTATGTCTGTGTGTCTGTGTTGTGTGTC	Chromosome 1	+	117641953	117642113	LINE
				Chromosome 1	+	30477429	30477466	SIMPLE
				Chromosome 1	+	9294085	9294148	SIMPLE
				Chromosome 1	+	15109760	15109794	SIMPLE
				Chromosome 1	+	113320956	113320980	SIMPLE
X17124.1				Chromosome 2	+	238126874	238127155	SIMPLE
				Chromosome 2	+	81459685	81459717	SIMPLE
				Chromosome 2	−	120372884	120372913	SIMPLE
				Chromosome 3	+	129370779	129370806	SIMPLE
				Chromosome 4	+	136831470	136831493	SIMPLE
				Chromosome 4	+	65516581	65516600	LTR
				Chromosome 5	−	38349981	38350374	SIMPLE
				Chromosome 6	−	8995374	8995414	SIMPLE
				Chromosome 6	+	166824634	166824662	SIMPLE
				Chromosome 6	+	161466706	161466730	SIMPLE
					−	73726269	73726328	SIMPLE
				Chromosome 7				
				Chromosome 8	+	141269742	141269778	SIMPLE
				Chromosome 9	−	136042391	136042690	LINE
				Chromosome 9	+	138142919	138142972	
				Chromosome 10	+	43224947	43225088	
				Chromosome 10	+	3808651	3808959	SIMPLE/LTR
				Chromosome 11	+	131867607	131867696	
				Chromosome 11	+	131681304	131681336	
				Chromosome 11	−	1491522	1491556	
				Chromosome 11	+	69064918	69064941	SIMPLE
				Chromosome 12	+	96976851	96976883	SIMPLE
				Chromosome 12	+	15253266	15253296	SIMPLE
				Chromosome 12	−	5481597	5481623	SIMPLE
				Chromosome 13	−	109571450	109571471	SIMPLE
				Chromosome 14	−	38591165	38591197	SIMPLE
				Chromosome 14	−	94651196	94651254	SIMPLE
				Chromosome 14	−	74427719	74427757	SIMPLE
				Chromosome 17	+	21737739	21737903	SIMPLE
				Chromosome 17	−	15465174	15465214	SIMPLE
				Chromosome 18	−	80862	81175	
				Chromosome 18	−	63198329	63198833	SIMPLE
				Chromosome 20	+	64153891	64153922	SIMPLE
				Chromosome 20	−	53433561	53433587	SIMPLE
				Chromosome 21	−	29213751	29214223	
				Chromosome X	−	1416564	1416597	SIMPLE
				Chromosome Y	−	1416564	1416597	SIMPLE
AY940476.1	1899	1921	ACAAAATTAATCCTAGAgACTGG	Chromosome 16	−	35270215	35270237	LTR
	9100	9133	ATATTTGAATGTATttAGAAAAATAAacAAATAG	Chromosome 1	−	71252085	71252108	Gene ZRANB2 antisense/Arthur1 hAT-Tip100
AY260554.1				Chromosome 10	+	18859243	18859275	LINE
				Chromosome 10	+	20553767	20553786	

* Bold capital is a matching base in VL30 sequences; the dot denoted a gap in the sequences.

## Data Availability

The data used to support the findings of this study (including transfection protocols and vector design) are included within the article. All other data used to support the findings of this study (including DNA sequences) are available from the corresponding author upon request.
